# Mineral profile of cocoa powder: Analytical approach in commercial samples

**DOI:** 10.1002/jsfa.70742

**Published:** 2026-05-24

**Authors:** Mateus Barbosa Silva, Herick Macedo Santos, Joane Cristina Costa Pereira, Heliara Caires Sousa, Raildo Mota de Jesus, Danilo Junqueira Leão, Leandro Soares Santos, Sibelli Passini Barbosa Ferrão

**Affiliations:** ^1^ Universidade Estadual do Sudoeste da Bahia – UESB Itapetinga Brazil; ^2^ Universidade Estadual de Santa Cruz – UESC Ilhéus Brazil

**Keywords:** ICP OES, chemometrics, nutritional quality, elements

## Abstract

**BACKGROUND:**

Cocoa powder is widely used in the food industry and has high nutritional value due to its composition, including minerals. The main factors related to the presence of minerals in cocoa powder are soil, processing, and cultivation practices. Given the high consumption of cocoa products and the need for information on their mineral composition, accurate assessments are important to ensure quality and consumer safety. In this study, the mineral content of commercial alkalized and natural cocoa powder samples from 12 countries was determined.

**RESULTS:**

Total mineral content was as follows: K, 16 921–63 731 mg kg⁻¹; P, 2129–9253 mg kg⁻¹; Mg, 2900–6183 mg kg⁻¹; Ca, 488–3985 mg kg⁻¹; Fe, 97.3–1154 mg kg⁻¹; Al, 5.65–917 mg kg⁻¹; Mn, 34.6–59.1 mg kg⁻¹; Cu, 21.9–48.1 mg kg⁻¹; Ni, 5.64–14.9 mg kg⁻¹; Cr, 0.479–18.5 mg kg⁻¹; and Cd, 0–1.30 mg kg⁻¹. Mineral content varied among samples, and hierarchical cluster analysis (HCA) grouped them into four clusters, as confirmed by principal component analysis (PCA). Sixteen samples exceeded the maximum cadmium limits.

**CONCLUSION:**

The proposed procedure was effective for determining minerals in cocoa powder. The samples showed wide variation across all elements and provided insights into the macro‐ and micronutrient composition of alkalized and natural cocoa powders. © 2026 The Author(s). *Journal of the Science of Food and Agriculture* published by John Wiley & Sons Ltd on behalf of Society of Chemical Industry.

## INTRODUCTION

Cocoa (*Theobroma cacao L*.) is an important commodity globally, with production primarily concentrated in Africa, notably in the Ivory Coast and Ghana. In the Americas, Brazil stands out as one of the largest producers, contributing around 22.6% of continental production and 4.56% of global production.^1^

Important commercial derivatives are produced from cocoa beans, including liquor, cocoa cake, cocoa butter, and cocoa powder. Among these derivatives, cocoa powder stands out for its use in various industries, including food, pharmaceuticals, and nutraceuticals, primarily due to its technological, sensory, and nutritional characteristics.^2^

Cocoa powder is derived from the processing of cocoa mass, paste, or liquor, in accordance with regulatory standards.^3^, ^4^ In the food industry, cocoa powder is a fundamental ingredient in confectionery, baking, frozen desserts, and beverage mixes.^5^, ^6^ It is a crucial component in the manufacturing of chocolate,^7^ a product widely consumed across demographics.^8^ The color, unique flavor profile, and chemical composition are primary factors influencing consumer preference.^2^

Commercial cocoa powder is available in natural or alkalized forms, depending on the production process. Alkalization is a step used to shape cocoa's technological characteristics, increasing solubility, intensifying color, and reducing bitterness.^9^ This process can also alter the mineral profile of the samples, as solutions such as potassium carbonate are used.

As cocoa powder is used in the formulation of various products, knowledge of its mineral content is important. Studies have reported the presence of essential and toxic elements (Ca, Mg, Mn, K, Fe, P, Cu, Cr, Ni, Na, S, Al, Pb, and Cd) in cocoa derivatives, including cocoa powder.^10^, ^11^, ^12^ Their concentrations may vary due to several factors,^13^ including soil, production processes, bean quality, climate, and plant genetic factors.^14^, ^15^, ^16^, ^17^

The mineral content is typically determined by spectroscopic methods, such as microwave‐induced plasma optical emission spectrometry (MIP OES),^18^ inductively coupled plasma optical emission spectrometry (ICP OES),^19^, ^20^ inductively coupled plasma mass spectrometry (ICP‐MS),^21^, ^22^ flame atomic absorption spectrometry (FAAS),^23^, ^24^ and graphite furnace atomic absorption spectrometry (GFAAS).^25^ Correct sample preparation procedures are mandatory to ensure that these analytical techniques yield reliable results.

Strategies for cocoa powder sample preparation for mineral analysis generally involve decomposition procedures using concentrated acids, which can be performed in closed microwave‐assisted systems^26^ due to the complexity of the matrix composition. However, the use of concentrated reagents presents drawbacks, including risks to operators and the environment, as well as potential equipment damage. The resulting digests also have high residual acidity, which may impair the atomization system.^27^ The use of diluted nitric acid solutions combined with hydrogen peroxide has been shown to be an effective alternative in microwave‐based procedures, as it allows satisfactory decomposition of organic matrices while reducing acid consumption and residual acidity during digestion.^21^, ^28^, ^29^, ^30^, ^31^, ^32^

In mineral analysis, particularly when large numbers of samples are evaluated, extensive datasets are generated, which may complicate the interpretation and discussion of the results. The use of exploratory chemometric tools such as principal component analysis (PCA) and hierarchical cluster analysis (HCA) is useful for aiding data interpretation and reducing dimensionality.^20^

Given recent efforts to minimize the use and generation of hazardous substances and to reduce pollutants, experimental design techniques have been applied widely to optimize sample preparation procedures in food analysis, in line with green chemistry principles.^33^ In this context, the aim of this work was to develop an analytical procedure for the determination of minerals in cocoa powder using microwave‐assisted digestion with dilute acid and ICP OES. A mixture design was used to optimize the composition of the oxidant solution. The procedure was applied to determine Ca, Mg, P, K, Ni, Cr, Cu, Mn, Al, Fe, Na, Zn, and Cd in commercial natural and alkalized cocoa powder samples from different countries.

## MATERIALS AND METHODS

### Sample collection

**Figure 1:**
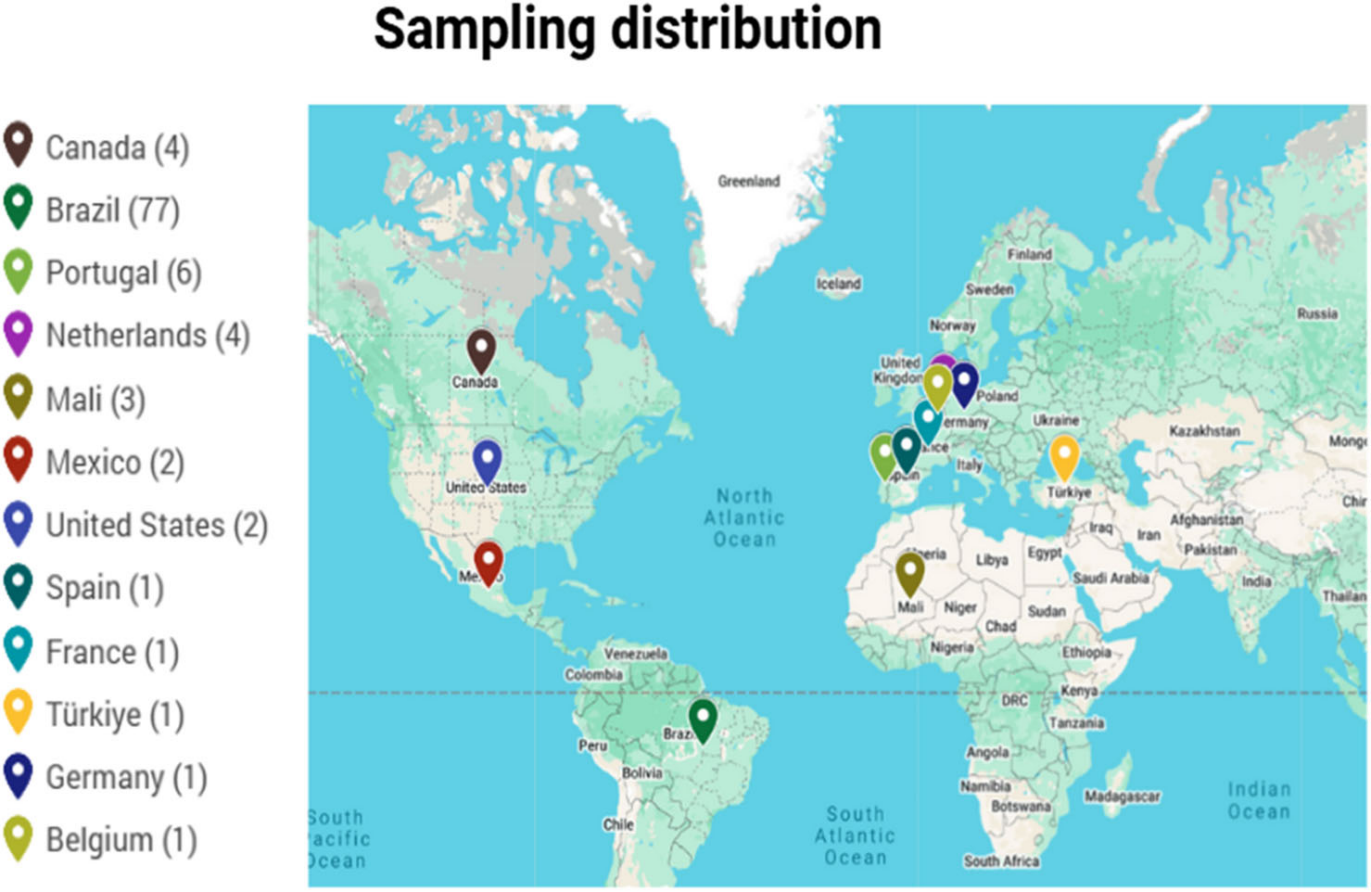
Distribution of cocoa powder samples by country.

A total of 103 commercial cocoa powder samples from different brands and geographical origins were collected between 2021 and 2023. Figure 1 shows the sample distribution. Among the samples, 51 were labeled as natural or alkalized, with 39 declared as alkalized and 12 as natural. The samples were kept in their original packaging (biaxially oriented polypropylene combined with metalized polyethylene) to ensure an effective barrier against light, moisture, and oxygen. All samples were properly sealed, stored frozen at −18°C, and analyzed within the expiration date indicated by the manufacturer. The packages were opened at the time of analysis, and 50 g of each sample was transferred from its original container to a polypropylene vial.

### Instrumentation

An inductively coupled plasma optical emission spectrometer, model 710‐ES (Varian, Mulgrave, Australia), equipped with a concentric nebulizer (Meinhard, Santa Clara, CA, USA) and a cyclonic nebulization chamber (Varian, Mulgrave, Australia) was used to determine Ca, Mg, P, K, Ni, Cr, Cu, Mn, Al, Fe, Na, Zn, Cd, and to estimate residual carbon content. The spectrometer was also equipped with a quartz torch (Varian, Mulgrave, Australia) in an axial configuration, and a solid‐state charge‐coupled device (CCD) detector. All analyses were conducted in accordance with the manufacturers’ instructions. Table 1 describes the instrumental conditions.

**Table 1:** Inductively coupled plasma optical emission spectrometry (ICP OES) instrumental operating conditions

Parameter	Configuration
Plasma power (kW)	1.35
Nebulizer pressure (kPa)	200
Plasma gas flow (L min⁻¹)	15.0
Auxiliary gas flow (L min⁻¹)	1.5
Pump speed (rpm)	15
Stabilization time (s)	15
Emission line (nm)	Mn 259.372 (II), Mg 279.800 (II), Ca 422.673 (I), K 769.897 (I), P 213.618 (I), Al 396.152 (I), Cr 267.716 (II), Cu 327.395 (I), Fe 238.204 (II), Cd 214.439 (II), Ni 231.604 (II), C 193.027 (I), Zn 213.857 (I), Na 589.592 (I)

Notes: (I) atomic emission line; (II) ionic emission line.

A Multiwave 5000 microwave oven (Anton Paar, Graz, Austria), equipped with a 41HVT rotor, was used for sample decomposition. An Elma ultrasonic bath (Singen, Germany) was used to eliminate volatile organic compounds (VOCs) during the determination of residual carbon.^34^

### Reagents and solutions

All solutions were prepared using high‐purity reagents. Ultrapure water was obtained from a Direct‐Q 3 UV Millipore purification system (Merck KGaA, Darmstadt, Germany), with a resistivity of 18.2 MΩ cm. Sodium hydroxide P.A. (Biotec, Paraná, Brazil) and citric acid monohydrate P.A. (Vetec, Rio de Janeiro, Brazil) solutions were used to determine residual acidity and residual carbon, respectively.

Pre‐distilled 65% nitric acid in a sub‐boiling distillation system, 30% Emsure hydrogen peroxide (H_2_O_2_) (Merck, Darmstadt, Germany), and ultrapure water were used for the microwave‐assisted decomposition procedure. A multi‐element standard solution (Specsol, São Paulo, Brazil) was used for ICP OES calibration. Analytical calibration curves were prepared from monoelemental standards (Specsol, São Paulo, Brazil) of 10 000 mg L^−1^ (Mg, K, and P) and 1000 mg L^−1^ (Ca, Ni, Cr, Cu, Mn, Al, Fe, Na, Zn, Cd). All glassware used was immersed in a 10% (v/v) HNO_3_ bath for 24 h and washed with ultrapure water.

### Microwave‐assisted decomposition

Sample preparation consisted of microwave‐assisted decomposition using a mixture of nitric acid (HNO₃), hydrogen peroxide (H₂O₂), and ultrapure water (H₂O). A total of 0.300 g of sample was weighed into modified polytetrafluoroethylene (PTFE‐TFM) vials using an analytical balance (Shimadzu AUW220D, Kyoto, Japan). Aliquots of HNO_3_ (7 mol L^−1^), H₂O₂ (30%), and H₂O were then added to the bottles, resulting in a final volume of 8 mL per experiment (Table 2).

**Table 2:** Mixture design matrix with pseudo‐components and actual values for the three components studied

Experiment	Pseudocomponents			Volume (mL)		
	HNO_3_	H_2_O_2_	H_2_O	HNO_3_ (7.0 mol L^−1^)	H_2_O_2_	H_2_O
1	0	0	1	3.00	1.00	4.00
2	1	0	0	6.00	1.00	1.00
3	0	1/3	2/3	3.00	2.00	3.00
4	2/3	1/3	0	5.00	2.00	1.00
5	0	1/6	5/6	3.00	1.50	3.50
6	1/2	0	1/2	4.50	1.00	2.50
7	1/3	1/3	1/3	4.00	2.00	2.00
8	5/6	1/6	0	5.50	1.50	1.00
9 (CP)	5/12	1/6	5/12	4.25	1.50	2.25
10 (CP)	5/12	1/6	5/12	4.25	1.50	2.25
11 (CP)	5/12	1/6	5/12	4.25	1.50	2.25

Abbreviation: CP, center point.

The vials were subjected to a heating program in accordance with the manufacturer's instructions,^35^ consisting of three stages: (i) 20 min heating ramp to 190 °C; (ii) 15 min plateau at 190 °C; (iii) cooling. After decomposition, the samples were transferred to 50 mL polypropylene tubes, and the volume was completed with ultrapure water up to 20 mL. The analytical blank was prepared in the same way, without the sample.

A reference experiment was performed in triplicate to evaluate the efficiency of sample preparation with dilute acid, using 5.0 mL HNO₃ (14 mol L^−1^) and 2.0 mL of H₂O₂ (30%).^34^, ^36^

### Multivariate optimization of the sample preparation procedure

A constrained mixture design was applied to optimize the composition of the oxidizing solution (HNO₃, H₂O, and H₂O₂), using a randomly selected sample. Statistica trial software was used to analyze the mixture design. The mixture composition followed the minimum and maximum restrictions established for each reagent: HNO_3_ (min.: 3 mL; max.: 6 mL), H₂O₂ (min.: 1 mL; max.: 2 mL), and H₂O (min.: 1 mL; max.: 4 mL), considering a maximum volume of 8 mL and the minimum concentration of HNO_3_ for an efficient decomposition process. A total of 11 experiments were established using the constrained mixture design, with three replicates at the global centroid to estimate experimental error. Table 2 shows the design matrix, including the pseudo‐components and the actual values for each experiment.

The best experimental condition was determined using multiresponse optimization, with the objective of simultaneously maximizing analytical signals and minimizing residual acidity (RA) and residual carbon content (RCC). The intensity signals obtained for each element were initially converted to a multiple response (MR) format.^37^ The responses were subsequently combined into a single response (SR), according to Eqn (1):

(1) $$\mathrm{SR}=\frac{\mathrm{MR}}{\left(\mathrm{RA}+\mathrm{RCC}\right)}$$

where *SR* = single response, *MR* = multiple response, *RA* = residual acidity, and *RCC* = residual carbon content.

### Determination of residual acidity and residual carbon

Residual acidity was determined by acid–base titration after microwave‐assisted digestion, in which an aliquot of the final digest was titrated using a standardized 0.0965 mol L^−1^ sodium hydroxide solution.^34^ Residual carbon content was determined using the method described by Gouveia *et al*.^38^ A calibration curve (0–100 mg L^−1^) was prepared using citric acid as the carbon source. An aliquot of the digest was diluted, sonicated for 3 min to remove volatile organic compounds, and subsequently analyzed by ICP‐OES under the conditions described in Table 1.

### Analytical parameters of the proposed procedure

The proposed procedure was evaluated using the following analytical parameters established by the Eurachem Guide and by Resolution No. 166 of the Collegiate Board from the Ministry of Health (ANVISA, BRASIL):^39^, ^40^ limit of detection (LOD), limit of quantification (LOQ), precision expressed as relative standard deviation (RSD), and accuracy. The concentration ranges for each element were: Mn, Ni, Cu (0.1 to 2 mg L^−1^); Cr, Cd (0.015 to 0.3 mg L^−1^) ; Al (0.4 to 2 mg L^−1^); Fe (0.1 to 4 mg L^−1^); Ca (3 to 50 mg L^−1^); Mg (1.5 to 15 mg L^−1^); P (2 to 20 mg L^−1^); K (5 to 100 mg L^−1^). To evaluate matrix effects, the slopes of the external calibration curve and the standard addition method were compared statistically.

Given the unavailability of certified reference material with a matrix similar to the samples studied, accuracy was evaluated through statistical comparison (*t*‐test) with the elemental content obtained from the proposed digestion procedure (dilute acid) and the reference procedure (using concentrated HNO₃),^21^ and by addition/recovery tests at two levels.

### Exploratory data analysis

Principal component analysis and hierarchical cluster analysis (HCA) were applied to identify patterns in the mineral composition of the analyzed samples. All analyte data were included in these analyses. For CA, the Euclidean distance matrix was used, and clusters were formed using Ward's method. The minimum number of groups was determined by evaluating the effect of adding each sample to a cluster using R^2^ values.

After CA, PCA was performed to identify which analytes characterized each group. A mean comparison test was then performed for each variable using Duncan's test at the 5% significance level. Statistical analyses were performed using SAS OnDemand for Academics (SAS, Cary, USA), and the PCA graph was generated using OriginPro 2023b (OriginLab Corporation, Northampton, USA), Learning Edition.

## RESULTS AND DISCUSSION

### Optimization of the sample preparation procedure

A mixture design with restrictions, using pseudo‐components HNO₃, H₂O₂, and H₂O, was employed to determine the optimal conditions for the decomposition of cocoa powder samples in a microwave oven. The restrictions were established because digestion does not occur in the absence of HNO₃. Thus, a minimum volume of 3 mL was established to ensure the necessary oxidizing potential of nitric acid. Maximum H_2_O_2_ volumes (2.0 mL) were also imposed to prevent pressure increases in the reaction flask and ensure operational safety.^41^

#### Determination of the optimal condition

The optimal experimental condition was determined using a mathematical tool capable of handling multiple responses by transforming the results of each experiment into a single response. The data were analyzed using Statistica software, in which the intensity signals for the elements, the RA and the RCC were used to define the optimal condition. The optimization criterion was to maximize the analytical signals and minimize RA and RCC. Accordingly, the experimental condition that combined the criteria to obtain the maximum emission intensities and the minimum values of RCC and RA was considered optimal. Table 3 reports the experimental responses.

**Table 3:** Responses of residual acidity, residual carbon content, analytical signal intensity and single response for mixture design

Exp.	Intensities													RA (mol L^−1^)	RCC (%)	MR	SR
	Mg	Ca	P	K	Ni	Al	Cr	Cu	Fe	Mn	Na	Zn	Cd				
1	75 377	417 271	10 483	3 024 980	854	38 384	2681	11 933	179 160	100 442	205 949	13 070	24.2	0.77	6.35	11.5	0.0119
2	72 510	423 072	9884	2 892 320	837	37 422	2675	11 895	176 445	98 577	204 271	12 655	25.2	1.81	8.58	11.25	0.0087
3	73 794	679 889	10 128	3 010 410	854	37 753	2549	11 627	178 937	99 504	203 168	12 692	17.4	0.97	8.10	11.97	0.0099
4	74 390	482 386	10 123	2 941 860	866	37 948	2911	11 709	180 942	99 802	203 772	12 775	35.2	1.64	9.20	11.99	0.0087
5	74 725	676 827	10 266	3 022 680	859	38 159	2607	11 726	179 988	100 783	266 321	12 781	14.6	0.92	8.59	12.12	0.0094
6	74 668	480 789	10 293	2 984 340	857	37 568	2606	11 641	179 136	100 135	239 847	12 763	13.9	1.21	9.27	11.43	0.0082
7	74 233	450 632	10 129	2 981 830	871	38 179	2682	11 790	180 542	101 236	266 229	12 927	19.4	1.33	9.77	11.51	0.0079
8	74 115	495 390	10 203	2 937 800	847	37 588	2719	11 997	179 126	100 176	210 652	12 731	15.9	1.69	10.42	11.37	0.0072
9	73 640	440 731	10 195	2 976 990	866	38 435	2677	12 110	181 558	101 272	206 765	12 731	14.7	1.35	9.67	11.22	0.0077
10	72 801	428 263	10 085	2 929 310	852	37 829	2621	11 774	179 320	99 954	238 353	12 793	17.8	1.33	8.95	11.23	0.0083
11	71 032	425 406	9828	2 860 720	822	36 563	2507	11 227	173 900	96 661	194 354	12 281	19.0	1.38	8.36	11.86	0.0085

Abbreviations: Exp., experiment; MR, multiple response; RA, residual acidity; RCC, residual carbon content; SR, single response.

An effective strategy for assessing digestion efficiency is to analyze the RCC,^42^ which is important for ICP OES analysis because it can interfere with spectral lines.^43^ Araujo *et al*.^44^ pointed out that carbon content below 13% does not interfere with elemental determination by ICP OES. The entire studied domain had an RCC content below 13%, with values between 6.35% and 10.42%.

Santos *et al*.^34^ developed and applied a microwave‐assisted decomposition procedure using diluted HNO_3_ and H₂O₂ to determine mineral content in guarana samples by ICP OES and reported RCC levels of 6.5% to 11.4% in the experiments. Oliveira *et al*.,^45^ when optimizing a decomposition method for chocolates, found an RCC value of 10% under the optimal experimental condition.

Another important point is residual acidity, which, depending on the concentration, can cause wear in the components of the equipment's sample introduction system.^41^ Experiments with low residual acidity do not require high dilution factors, enabling efficient determination of elements at low concentrations. In the present study, residual acidity ranged from 0.77–1.81 mol L^−1^, and acidity decreased as smaller volumes of nitric acid were used.

**Figure 2:**
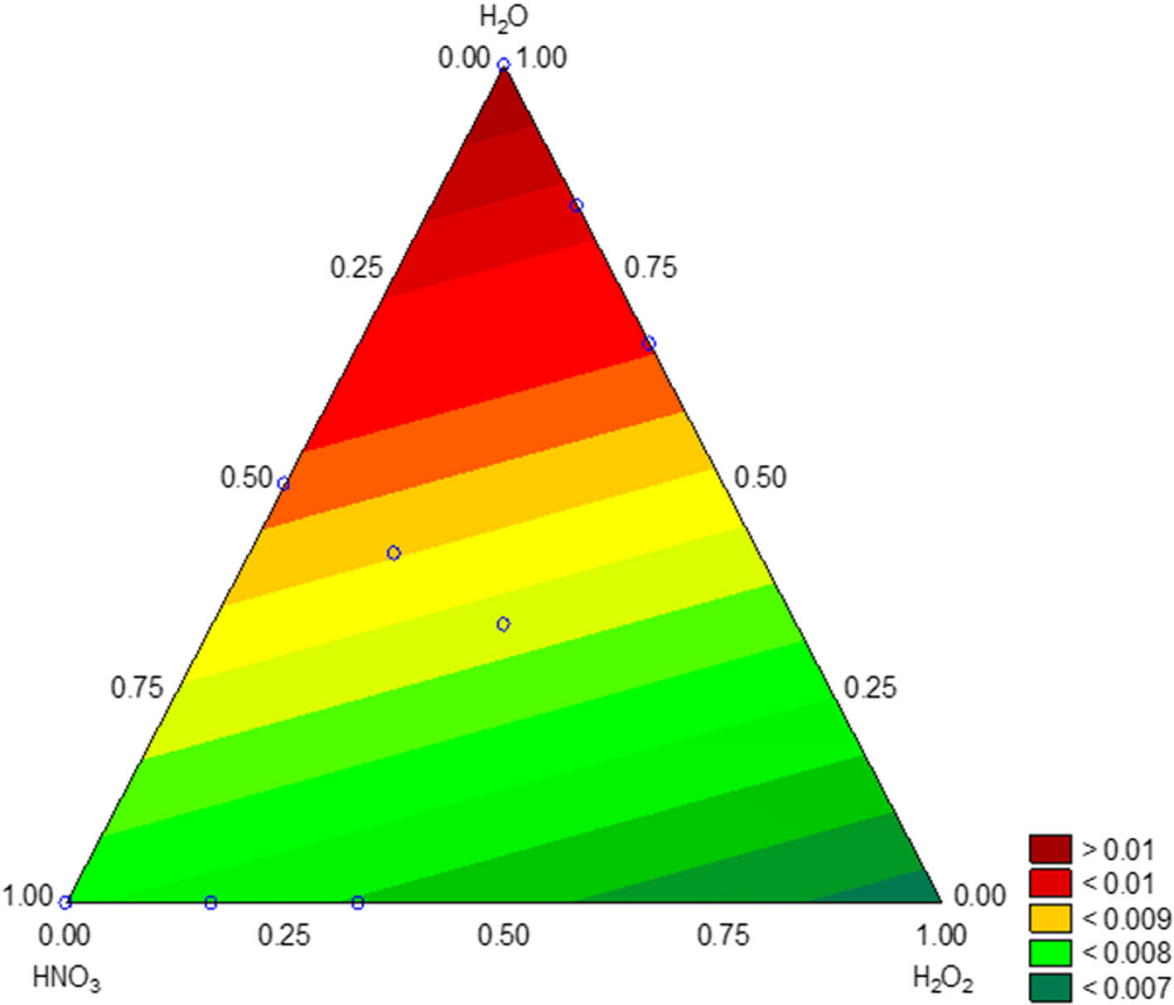
Contour plot for the pseudocomponents of the mixture (HNO₃, H₂O, and H₂O₂).

The results show that the signal intensities of all elements were similar, except for Ca, which showed increased intensity in experiments 3 and 5 (Table 3). As for the other parameters evaluated, experiment 1 showed the best results, with the lowest residual acidity concentration (0.77 mol L^−1^), lowest residual carbon content (6.3%), and the best single response. The contour plot for the studied domain showed a triangular region (Fig. 2). The optimal mixture composition corresponded to experiment 1, where 3.0 mL of HNO₃ (7.0 mol L^−1^), 1.0 mL of H₂O₂ (30% m/m), and 4.0 mL of H₂O were used, resulting in an HNO₃ solution of approximately 3.0 mol L^−1^.

The contour plot (Fig. 2) shows that the region with the best responses corresponds mainly to the interaction between H_2_O_2_ and H_2_O. The condition that generated the best response corresponds to the experiment that used the largest volume of water used in the experiments and the smallest volume of the other components (experiment 1).

#### Evaluation of the optimized microwave‐assisted digestion

Table 4 shows the analytical parameters for the proposed procedure. The LOD and LOQ values were satisfactory for the intended application. For the RSD, all values were less than 10% for all elements, demonstrating the procedure's satisfactory precision. Accuracy was evaluated by comparing the results from the proposed microwave‐assisted digestion using dilute acid with those from the reference procedure. As Table 4 shows, the total content of all elements did not differ significantly at the 95% confidence level. In the addition/recovery test (Table 5), satisfactory recoveries were observed for all elements (82% to 110%), demonstrating the proposed procedure's acceptable accuracy. Table 6 also shows that no evidence of matrix effects was observed; that is, the ICP OES sensitivity was not significantly altered when using the standard addition method. It was therefore concluded that the proposed procedure could be applied to the determination of minerals in cacao powder samples.

**Table 4:** Validation parameters of the decomposition procedure for cocoa powder samples: limit of detection (LD), limit of quantification (LQ), precision and accuracy

	LD		LQ		Precision	Accuracy	
Analyte	mg L^−1^	mg kg ^−1^	mg L^−1^	mg kg^−1^	RSD (%)	Proposed procedure (mg kg ^−1^)	Reference procedure (mg kg ^−1^)
Cd	0.0008	0.053	0.0028	0.187	2.36	<LD	<LD
Cr	0.0010	0.067	0.0035	0.233	3.01	7.11 ^a^ ± 0.53	7.89 ^a^ ± 0.13
Ni	0.0020	0.133	0.0065	0.433	2.03	8.97 ^a^ ± 0.56	9.84 ^a^ ± 0.26
Cu	0.0020	0.133	0.0067	0.447	2.27	40.6 ^a^ ± 2.1	43.0 ^a^ ± 0.92
Mn	0.0013	0.087	0.0044	0.293	1.54	49.0 ^a^ ± 3.2	51.9 ^a^ ± 0.93
Al	0.0151	1.01	0.0502	3.35	3.96	160 ^a^ ± 10	155 ^a^ ± 2
Fe	0.0132	0.880	0.0441	2.94	2.00	519 ^a^ ± 35	562 ^a^ ± 8
Ca	0.0973	6.49	0.3244	21.67	1.62	1534 ^a^ ± 74	1642 ^a^ ± 63
Mg	0.0507	3.38	0.1692	11.28	2.57	5841 ^a^ ± 352	5872 ^a^ ± 166
P	0.0186	1.24	0.0622	4.15	2.60	7772 ^a^ ± 457	7677 ^a^ ± 231
K	0.0325	2.17	0.1083	7.22	2.58	31 600 ^a^ ± 2154	30 461 ^a^ ± 789
Na	0.0024	0.160	0.0080	0.533	5.48	252^a^ ± 16	235^a^ ± 6
Zn	0.0083	0.553	0.0276	1.84	1.83	80.2^a^ ± 4.6	83.5^a^ ± 2.6

RSD, relative standard deviation.

**Table 5:** Recovery at two concentration levels: initial concentration, responses to additions 1 and 2 (mean ± standard deviation, *n* = 3)

Analyte	Initial concentration (mg L^−1^)	AD 1 (mg L^−1^)	Results AD 1	Rec (%)	AD 2 (mg L^−1^)	Results AD 2	Rec (%)
Cd	0.018 ± 0.0002	0.05	0.071 ± 0.001	104 ± 3	0.1	0.125 ± 0.0006	106 ± 1
Cr	0.10 ± 0.01	0.1	0.20 ± 0.01	98.1 ± 2.6	0.2	0.31 ± 0.01	100 ± 8
Ni	0.13 ± 0.01	0.1	0.22 ± 0.00	92.3 ± 8.8	0.2	0.32 ± 0.00	92.7 ± 6.2
Cu	0.59 ± 0.04	0.5	1.05 ± 0.02	97.5 ± 8.3	1	1.60 ± 0.02	101 ± 6
Mn	0.71 ± 0.05	0.5	1.16 ± 0.01	88.4 ± 7.3	1	1.67 ± 0.04	95.4 ± 9.1
Al	2.09 ± 0.12	2	3.89 ± 0.10	95.5 ± 2.1	4	5.99 ± 0.10	97.6 ± 5.4
Fe	7.67 ± 0.40	5	11.9 ± 0.28	91.6 ± 5.8	10	16.5 ± 0.41	88.6 ± 9.2
Ca	22.2 ± 1.14	10	30.4 ± 0.54	82.4 ± 9.6	20	42.0 ± 1.15	92.0 ± 0.6
Mg	8.53 ± 0.53	10	18.7 ± 0.23	102 ± 6	20	30.6 ± 1.09	110 ± 8
P	11.5 ± 0.69	10	21.5 ± 0.12	99.4 ± 7.7	20	33.3 ± 1.03	109 ± 8
K	48.9 ± 3.23	10	57.4 ± 0.86	82.0 ± 7.2	20	70.9 ± 2.32	110 ± 15
Na	3.93 ± 0.25	10	13.7 ± 0.29	97.8 ± 1.9	20	24.2 ± 0.53	102 ± 4
Zn	1.22 ± 0.07	1	2.18 ± 0.04	101 ± 5	2	3.31 ± 0.05	104 ± 6

AD, addition; Rec, recovery.

**Table 6:** Slopes for external calibration curves and standard addition

Analyte	External calibration curve		Curve with added standard	
	Slope	SD	Slope	SD
Cd	15986^a^	162	15 350^a^	679
Cr	26 388^a^	249	25 977^a^	149
Ni	7062^a^	153	6702^a^	99
Cu	21 383^a^	381	20 714^a^	87
Mn	130 463^a^	1043	128 133^a^	1496
Al	1031^a^	11	1007^a^	40
Fe	24 837^a^	326	24 098^a^	92
Ca	16 236^a^	519	15 382^a^	792
Mg	4778^a^	11	4663^a^	62
P	470^a^	23	430^a^	5
K	41 443^a^	524	41 513^a^	2743
Na	214 076^a^	5679	217 151^a^	2221
Zn	11 197^a^	199	10 875^a^	443

*Note*: Means with the same letter on the same line are not significantly different at 5%. SD, standard deviation.

### Application in commercial cocoa powder samples

The proposed method was applied to determine the Ca, Mg, P, K, Ni, Cr, Cu, Mn, Al, Fe, and Cd content in commercial cocoa powder samples. The results for each sample are available in the Supporting Information. Table 7 reports the mean values, the medians, and range (minimum and maximum values) for all minerals found in commercial cocoa powder samples.

**Table 7:** Concentration of minerals in commercial cocoa powder samples (mean values, medians, and range)

Analyte	Mean (mg kg^−1^)	Median (mg kg^−1^)	Range (mg kg^−1^)
Cd	0.209	0.000	0–1.30
Cr	5.25	4.36	0.479–18.5
Ni	8.58	8.42	5.64–14.9
Cu	36.1	37.2	21.9–48.1
Mn	46.5	45.7	34.6–59.1
Al	215	166	5.65–917
Fe	495	428	97.3–1154
Ca	1757	1518	488–3985
Mg	5222	5314	2900–6183
P	6129	6618	2129–9253
K	36 000	34 833	16 921–63 731

The cocoa powder samples showed a wide range of mineral content, demonstrating great variability. The total mineral content followed the order: K > P > Mg > Ca > Fe > Al > Mn > Cu > Ni > Cr > Cd. These results may be related to several factors, including cocoa variety, processing method, soil composition, and bean quality. Millena *et al*.^12^ observed a decrease in mineral concentration over the course of cocoa fermentation, attributed to the leaching of soluble minerals, suggesting that the fermentation process may also influence the concentration of elements in processed cocoa.

The minerals with the highest content in the cocoa powder samples were found to be K, P, Mg, and Ca, as evidenced in other studies such as those by Bertoldi *et al*.^46^ and Grembecka and Szefer.^47^ Febrianto and Zhu^10^ reported the same minerals as predominant in cocoa beans, with relatively lower levels. This can be explained by processing stages such as partial fat removal, element concentration, and alkalization, which can influence the levels of some of these minerals.

Total Cu content ranged between 21.9 and 48.1 mg kg^−1^, Mn from 34.6 to 59.1 mg kg^−1^, and Cr from 0.479 to 18.5 mg kg^−1^. These variations can be attributed to sampling variability across different producing regions, to tropical soils that may enrich certain minerals, and processing differences, as the method used to concentrate and obtain the powders can alter the minerals after the butter is removed.

Similar levels were found by Febrianto and Zhu^10^ in cocoa beans for Cu (22.1–33.9 mg kg^−1^), Mn (23.4–54.4 mg kg^−1^), and Cr (1.44–7.81 mg kg^−1^). The similar content of these elements in beans and in the samples studied suggests that these minerals are not susceptible to concentration or dilution during cocoa processing.

Aluminium concentrations ranged from 5.65 to 917 mg kg^−1^ and Fe concentrations ranged from 97.3 to 1154 mg kg^−1^. The variation in the values found may be due to the use of equipment and utensils made of these materials in cocoa processing, which could lead to their migration into the product. Given cocoa powder's role as an ingredient in many daily products, the concentrations of Al and Fe, as well as other minerals, may have cumulative effects. The levels of Al, Ni, and Cd are a concern because these elements pose potential health risks. In the samples studied, significant variations in the levels of these minerals were observed, with some samples exhibiting high concentrations. The European Food Safety Authority (EFSA) expressed concerns in one of its reports about the relationship between aluminum and neurodegenerative diseases.^48^ Nickel is an element that exhibits biological activity and, in the short term, can cause allergic reactions. It can result in organ damage due to its cumulative effect.^17^

The presence of Cd is a concern in food samples because it is toxic even at low concentrations, which is why regulatory agencies establish maximum tolerable limits (MTLs) in various foods. De Oliveira *et al*.^15^ point to growing concerns about the levels of this element in cocoa and its derivatives. For cocoa powder, according to EU regulations, the MTL is set at 0.60 mg kg^−1^.^49^ In this study, 15.5% of the samples analyzed had values above this limit. Other legislation for chocolate and cocoa‐based products with more than 40% cocoa establishes an MTL of 0.30 mg kg^−1^,^50^, ^51^ and considering this legislation, 24.3% of the samples were non‐compliant for this element.

The toxicity of Cd is well documented, as this element is associated with nephrotoxicity, carcinogenicity, and bone demineralization. Cadmium accumulates in the human body due to its long biological half‐life (10–30 years), particularly in the kidneys and liver, and poses greater risks when cocoa‐derived products are a significant part of the diet. Cocoa is also a source of Cd because *Theobroma cacao* can absorb this metal from the soil, with higher concentrations reported in Latin American cocoa than cocoa with other origins.^52^, ^53^

#### Exploratory data analysis

**Figure 3:**
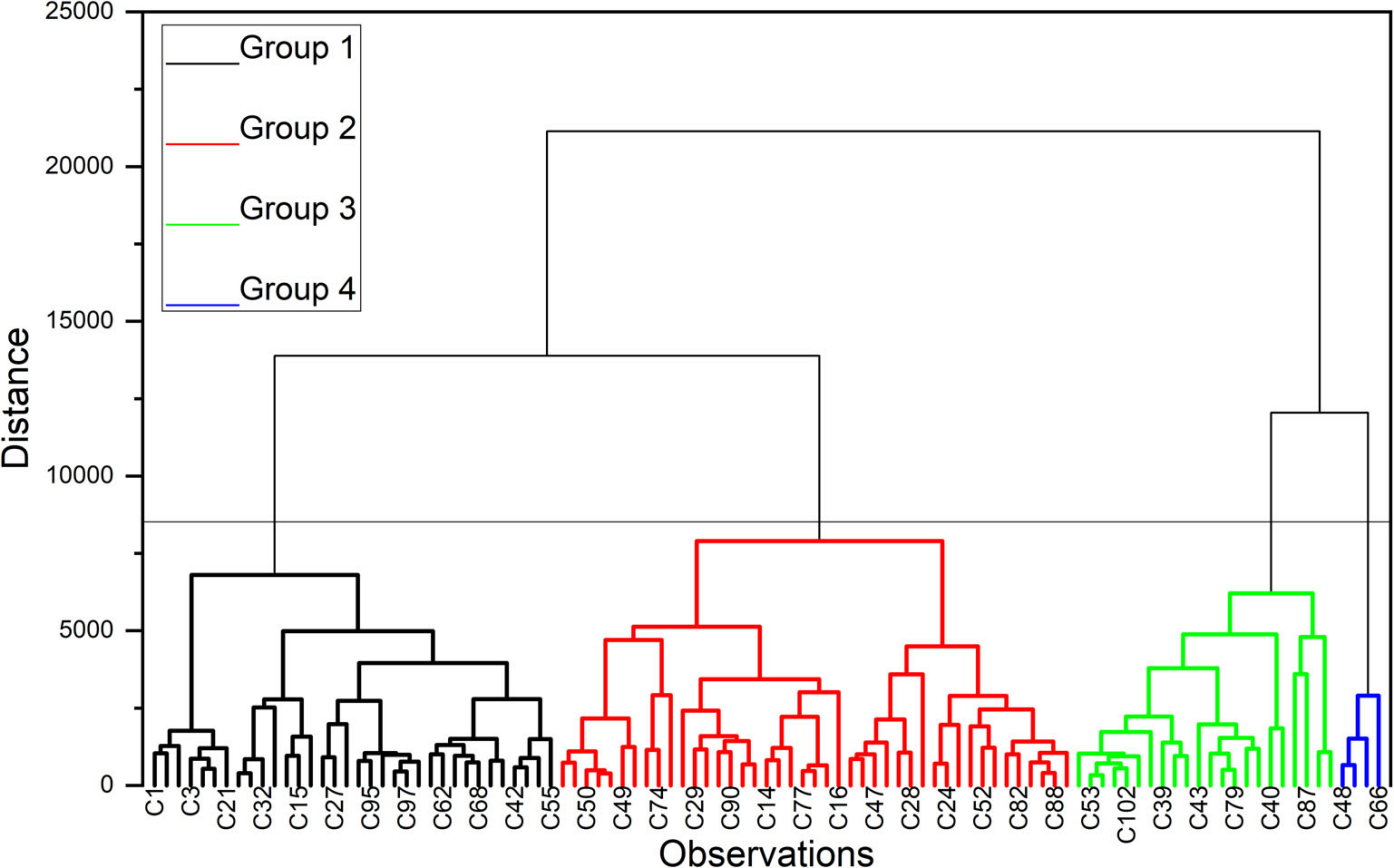
Dendrogram of the hierarchical cluster analysis of cocoa powder samples, showing the cutoff point used to define four groups.

Hierarchical cluster analysis made it possible to identify groups within the sample set with similar composition. Based on the dendrogram analysis (Fig. 3), four groups were selected. Groups 3 and 4, consisting of 19 and eight samples, respectively, shared common characteristics in terms of origin, as all samples in these groups were of Brazilian origin. Group 1 consisted of 44 samples from Brazil (34), Netherlands (2), Mexico (2), Canada (1), Portugal (1), Germany (1), the USA (1), Turkey (1), and France (1). Group 2 included 32 samples: Brazil (16), Portugal (5), Mali (3), Canada (3), the Netherlands (2), the USA (1), Belgium (1), and Spain (1). The sample distribution across countries was not uniform, making it impossible to evaluate grouping by geographical origin. Although Brazilian samples predominated, they did not show similarity in their mineral profiles.

As for the type of cocoa, alkalized (A), natural (N), or no information (NI) on the label, the distribution in the groups was as follows: group 1: 23 A, 4 N and17 NI; group 2: 7 A, 5 N and 20 NI; group 3: 5 A, 2 N, and 12 NI; group 4: 4 A, 1 N, and 3 NI. Group 1 accounts for 58.97% of the alkalized samples in the sample set, which may be a distinguishing factor for this group. As for the groups, no common characteristics were observed regarding the type of cocoa powder.

**Figure 4:**
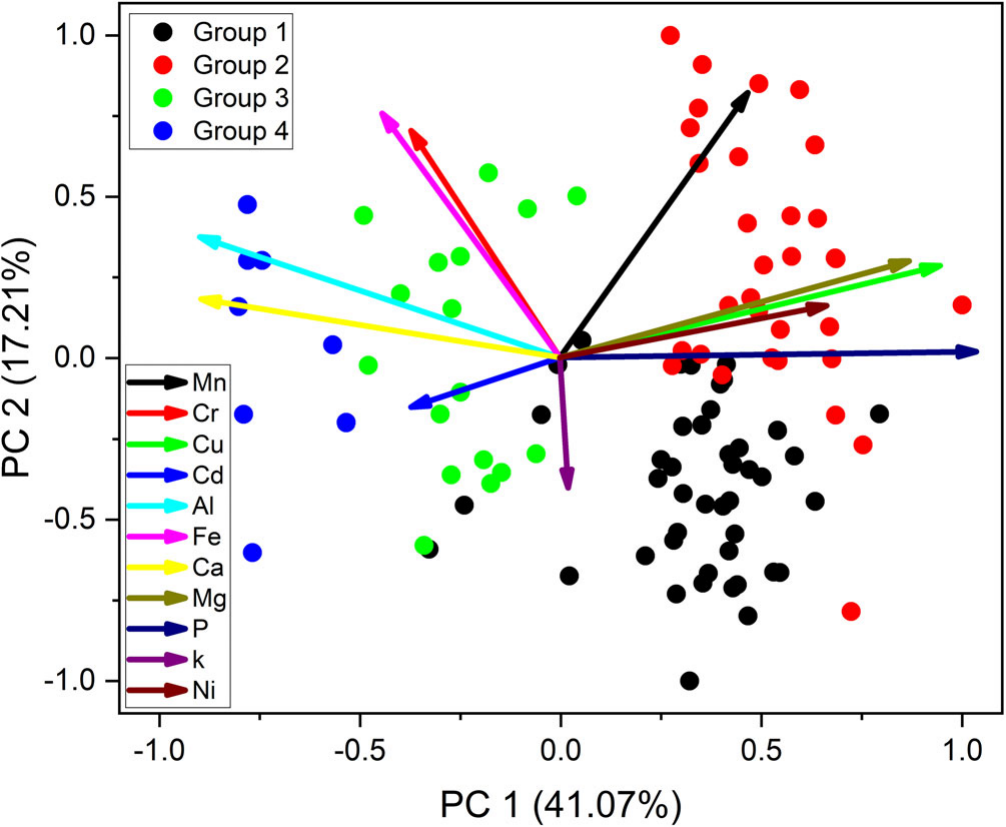
Scores plot of PC1 and PC2 for cocoa powder samples.

Principal component analysis was used to identify variables characterizing each group. Only two components were required to observe sample dispersion (Fig. 4). Group 1, is in the fourth quadrant and is characterized especially by high K, P, Ni, Cu, and Mg content. The higher K content in this group may be due to alkalized samples, which represent 52.27% of the group. Potassium carbonate (K_2_CO_3_) is one of the most commonly used agents in the cocoa alkalization process.^54^ This mineral may be transferred to the product during this process.

High levels of Ni, Cu, Mn, Fe, Mg, and P characterized group 2. However, there was no uniformity in the origin or type of cocoa in these samples that could explain their relationship, due to the high sample variability across eight countries.

Groups 3 and 4 share the common characteristic of originating solely from Brazilian samples. However, in terms of composition, group 3 is characterized by high Cr values and group 4 by high Cd, Al, and Fe content. Both groups have lower Ni, Cu, Mg, and P content than the others. One factor that may explain the wide variation in the mineral content of the samples is the mixing of cocoa bean varieties from different regions for the production of cocoa powder.

## CONCLUSION

This study proposed an analytical procedure for assessing mineral content in cocoa powder, enabling quantification of the elemental profile in samples from different countries. The optimized procedure can aid in monitoring potentially toxic elements and characterizing cocoa powder, and may be applied to future research on authenticity, traceability, and related areas.

The proposed microwave‐assisted procedure using diluted acid and H_2_O_2_ proved efficient for decomposing the samples and making the minerals contained in cocoa powder available for subsequent determination by ICP OES without substantial losses. The procedure yielded satisfactory analytical parameters (LOD, LOQ, precision, and accuracy). The use of a dilute oxidant solution (only 3.0 mol L^−1^ of HNO_3_) for sample preparation of cocoa powder is advantageous because it makes the analysis more feasible by reducing reagent costs and posing fewer risks to the operator and the equipment. Thus, the procedure described here may represent a viable alternative for the routine determination of minerals in cocoa powder.

The most abundant minerals in cocoa powder were K and P. Total K content ranged from 16 921 to 63 731 mg kg⁻¹, whereas total P content ranged from 2129 to 9253 mg kg⁻¹. The total content of Cd (0–1.30 mg kg^−1^), Ni (5.64–14.9 mg kg^−1^), and Al (5.65–917 mg kg^−1^) indicates the importance of considering appropriate management or remediation strategies to reduce the levels of these potentially toxic elements. In general, the mineral content of cacao powder samples showed wide variation, likely due to regional variability.

The results of this study provided important insights into the macro‐ and micronutrient composition of cocoa powder, particularly for Cd, for which 15.5% of samples exceeded regulatory limits. However, further studies on bioaccessibility and bioavailability are needed to assess the nutritional quality and food safety of cacao powder samples comprehensively.

## CONFLICT OF INTEREST

The authors declare no conflict of interest.

## Supporting information


**Data S1:** Supporting Information.

## Data Availability

Research data are not shared.
